# Current Challenges in Glioblastoma: Intratumour Heterogeneity, Residual Disease, and Models to Predict Disease Recurrence

**DOI:** 10.3389/fonc.2015.00251

**Published:** 2015-11-16

**Authors:** Hayley P. Ellis, Mark Greenslade, Ben Powell, Inmaculada Spiteri, Andrea Sottoriva, Kathreena M. Kurian

**Affiliations:** ^1^Brain Tumour Research Group, Institute of Clinical Neurosciences, University of Bristol, Bristol, UK; ^2^Bristol Genetics Laboratory, North Bristol NHS Trust, Bristol, UK; ^3^School of Mathematics, University of Bristol, Bristol, UK; ^4^Centre for Evolution and Cancer, The Institute of Cancer Research, London, UK

**Keywords:** GBM, intratumor heterogeneity, neural networks, residual disease, Bayesian models

## Abstract

Glioblastoma (GB) is the most common primary malignant brain tumor, and despite the availability of chemotherapy and radiotherapy to combat the disease, overall survival remains low with a high incidence of tumor recurrence. Technological advances are continually improving our understanding of the disease, and in particular, our knowledge of clonal evolution, intratumor heterogeneity, and possible reservoirs of residual disease. These may inform how we approach clinical treatment and recurrence in GB. Mathematical modeling (including neural networks) and strategies such as multiple sampling during tumor resection and genetic analysis of circulating cancer cells, may be of great future benefit to help predict the nature of residual disease and resistance to standard and molecular therapies in GB.

## Introduction

Glioblastoma (GB) accounts for approximately 65% of all primary brain tumors and is characterized by low survival, with only 10% of patients surviving 5 years (1). It is for this reason that there is an urgent need for a deeper understanding of the genetic pathways behind the development of GB, as well as its maintenance and progression. The increased use of novel technologies to identify critical genomic alterations in each individual tumor may help identify specific predictive biomarkers, leading to a personalized treatment approach (2).

The emergence of molecular biomarkers in brain tumors has been of great benefit both diagnostically and for stratifying therapies (3). Routine tests for both predicting prognosis and stratifying patients for therapies now include assessing BRAF and IDH1/2 mutations, as well as MGMT promoter methylation status to predict response to temozolomide – the current gold standard in GB treatment (Stupp protocol) (4–6). Inactivation of the MGMT enzyme by promoter hypermethylation can be a positive predictor of response to temozolomide due to the resultant inability of the MGMT enzyme to remove alkyl groups from DNA (5). However, treatment of GBs that harbor the hypermethylated MGMT with alkylating agents introduces thousands of new mutations, culminating in a highly mutable phenotype via loss of DNA mismatch repair or other repair mechanisms (7).

Following the study by the TCGA consortium (8), Verhaak et al. described a gene expression-based classification of GB into four molecular subgroups based on the previously elucidated expression of signature genes (as shown in Table 1) (9). The group obtained microarray data for 601 genes from 116 core samples used in the recent TCGA project, in addition to 73 samples previously described (9). In this study, different subgroups of GB appeared to have different responses to therapy (*p* = 0.02) (9). However, the identification of different subgroups of GB does not account for intratumour heterogeneity within the primary tumor nor for the tumor clonal evolution during the course of the disease and in response to treatment (10).

**Table 1:** **Gene expression in hypothesized glioma subclasses**.

Classical	Mesenchymal	Proneural	Neural
chr7 ↑/chr10 – (100%)	NF1 ↓ (53%)	PDGFRA ↑	NEFL
EGFR ↑ (97%)	NF1 ↓/PTEN ↓ (86%)	IDH1	GABRA1
EGFRvIII (55%)	MET	TP53 +/−	SYT1
CDKN2A −/−	CHI3L1	TP53 LOH	SLC12A5
NES ↑/Notch ↑	CD44 ↑/MERTK ↑	chr7 ↑/chr10 – (54%)	
SHH signaling ↑	NFκB signaling ↑	OLIG2 ↑ (CDKN1A ↓)	
		SOX	
		TCF4	

*The table shows changes in gene expression and LOH/deletions in genes in the four subclasses of glioma proposed by Verhaak et al. (9). The changes in gene expression that are characteristic of each subtype are highlighted in each column, and the percentage of cases that express these changes are present for the most ubiquitously expressed genes*.

This review will discuss the problems presented by genomic and transcriptomic intratumour heterogeneities in tumor evolution and treatment resistance, as well as the effect of residual disease from cells in the subependymal zone. Examples of mathematical models including neural networks and Bayesian models will then be outlined, and their potential for application to the field of brain tumor genetics and disease prediction will be evaluated. Overall, this review aims to address the utility of mathematical models as tools for predicting intratumour heterogeneity, disease progression, and treatment resistance in GB using the next-generation sequencing techniques.

## Intratumour Heterogeneity and Treatment Resistance

Comparative analysis of subsections from the same tumor mass using microdissection has been used to illustrate intratumor heterogeneity since the late 1990s, though current advances in the next-generation sequencing and single-cell analysis have only recently begun to identify possible mechanisms with which intratumour heterogeneity is linked to treatment resistance (11, 12). It is becoming increasingly recognized that one of the primary mechanisms related to treatment failure and tumor recurrence in GB may be intratumour molecular heterogeneity, though the pathways that underlie this process require further elucidation, as summarized in the recent review by Parker et al. (13–18).

In a recent study, we examined intratumour heterogeneity across 11 GBs at a genomic and transcriptomic levels by comparing superficial and deep tumor fragments from the same patient (10). Interestingly, differences in gene expression and copy numbers meant that fragments from the same patient were often categorized into distinct subclasses of GB according to the Verhaak criteria (10).

**Figure 1:**
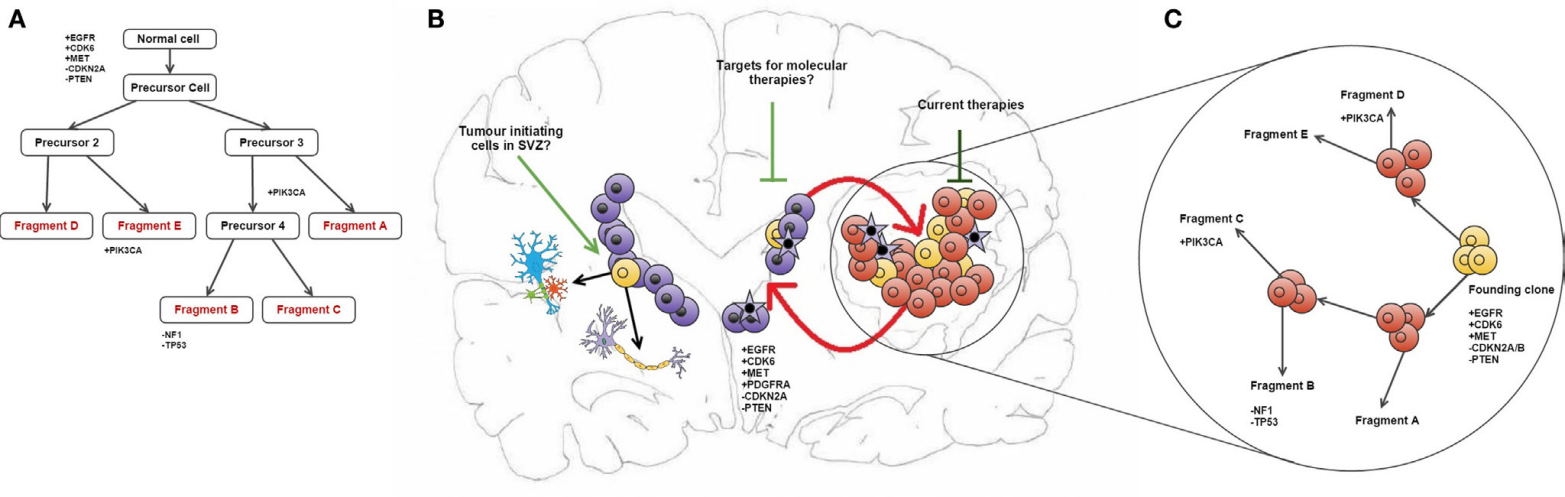
**Intratumour heterogeneity in the evolution of GB**. **(A)** Phylogenetic reconstruction of a GB case based on copy number alterations. **(B)** It has been hypothesized that TICs reside in the SEZ and can contribute to tumor maintenance (19). These cells (shown in purple) may be potential new targets for molecular therapies in GB. Also shown is the maintenance of the tumor bulk by cells in the SEZ containing the same mutations as described in the phylogenetic tree, and how current therapies target the tumor bulk (shown in red). **(C)** combines **(A,B)** to give an overview of tumor evolution in this case across time and in physical location. The case described in all parts of this diagram and all corresponding genetic information was obtained from Sottoriva et al. (10). **(B)** is adapted from the review by Goffart et al. (20).

Some of the copy number aberrations (CNAs) occurring in the key pathways involved in gliomagenesis (p53, Rb, and RAS/RTK/PI3K) were found to be heterogeneous within tumor samples, as exemplified by PDGFRA (see in Figure 1). Although the genetic signature of fragments from the same patient shows a level of similarity that indicates clonal expansion from a common ancestor during gliomagenesis, great variation in CNAs was found between fragments reflecting the underlying tumor evolution (10). The methodology employed in this study has allowed both spatial and temporal analyses of intratumoural heterogeneity in GB at the genotype level (copy number) as well as the cellular phenotype (dictated by gene expression levels) (see Figure 1) (10).

Recent technological advances have also allowed the analysis of cancer genetics to be conducted on the single-cell level. Patel et al. recently profiled 430 cells from five primary GBs and found that individual cells could be classified as different types of GB according to the TGCA classification scheme (21). This may be indicative of a problem with the clinical efficacy of molecular therapies, as significant intercell variability was found between different splice variants and levels of expression of RTKs, among other signaling molecules, often used as therapeutic targets (21). This observation of heterogeneous amplification appears several times across the literature, most commonly studied in EGFR, PDGFRA, and MET due to their significance in GB (22, 23).

Similar methods, including ultradeep sequencing, were used by Nickel et al. to analyze the mutational heterogeneity across time in recurrent GB as a part of a case study (24). Candidate mutations were identified and independently validated to produce a coherent mutational landscape of CNVs across primary GB and recurrences, citing mutation calling accurate to a 10% frequency detection threshold (24). The total gene coverage varied substantially in this study (sample mean range 81–145 across interrogated exons); however, intragene coverage was consistent, and a 10% threshold has also been cited in other similar studies (24, 25). Furthermore, studies of this nature can be rare due to the difficulty of obtaining tissue that has been adequately preserved, and the necessity for such high levels of coverage required to detect somatic mutations in such small subsections of tissue.

### Genomic Variation During Tumor Evolution and Recurrence

The variation in CNA as described over time in tumor development is summarized in **Table 2**. It appears that early in the growth phase of the tumor, the CNAs are most frequently localized to chromosomes 7 and 10, as these are the locations of the driver genes EGFR, MET, PTEN, and CDK6 (10). Copy number deletions on chromosome 9 (CDKN2A/B locus) and the 10p12 locus were also described during the common early phase (10). The middle shared phase of development consists of an accumulation of chromosome 7 and 19q12/13 amplifications, as well as focal amplifications of PDGFRA; during this stage, deletions occur almost exclusively at the PTEN locus on chromosome 10, which leads to further deregulation (10). The late phase (inferred by the presence of unique variants) harbors a peak of variance at the GLUT9 gene on chromosome 4p16 which has previously been described as a regulator of cancer cell glucose metabolism (26), but the most significant observation was that late-phase mutations were more widely scattered across the genome than the previous proposed stages (10).

**Table 2:** **A summary of copy number variations during tumor progression**.

Early phase (common mutations)		Middle phase (shared mutations)		Late phase (unique mutations)	
Gain/amplification	Deletion	Gain/amplification	Deletion	Gain/amplification	Deletion
EGFR	CDKN2A/B	chr7	chr10	GLUT9	
CDK6	PTEN	19p12/13	(PTEN)	PDGFRA	
MET		PDGFRA			

*The patterns in genetic variance over time described in this table were obtained from the study by Sottoriva et al. (10)*.

More recently, multiregion sequencing efforts have elucidated on the evolution of recurrence in GB by profiling multiple regions of the primary tumor as well as multiple regions of the recurrent mass. These have identified mixed evolutionary patterns between patients, where some patients were characterized by linear evolution and others by divergent evolution (27, 28). These next-generation sequencing efforts provide crucial evidence on the dynamics of both the intra- and interpatient genomic heterogeneities in GB-driving recurrent disease.

Consequently, studies using tissue obtained from single sample methods may be at risk of sampling bias, as evidenced by the significant genetic variance between samples from the same tumor mass (19). Thus, it may also be important to factor intratumour heterogeneity and its subsequent effects on tumor subpopulations into any molecular guidelines that may be used for patient stratification for targeted molecular therapies (19). This could be furthered by analyzing the evolutionary dynamics of genetic variance within a larger population, using paired primary and recurrent tumor samples to identify variance (19). Future studies should also attempt to further elucidate the cellular interactions of tumor fragments within the microenvironment, as this would shed further light on the intricate pathways that facilitate gliomagenesis (19).

## Identifying Residual Disease in the Subependymal Zone

Another significant challenge in the treatment of GB may be the presence of residual disease in the subependymal zone (SEZ). In the adult brain, neurogenesis occurs in the SEZ, supplying the cortex and corpus callosum with glial cells and the olfactory bulbs with neurons (29). In a previous study, we identified malignant cells in the SEZ of GB patients, which could contribute to disease recurrence by harboring the tumor-initiating cells (TICs) that comprise residual disease (see Figure 1) (19). The TICs were isolated using a cell-surface marker-independent approach, as CD133 and CD15 glioma stem cell markers have been proven unreliable (30). It is not yet known whether the TICs in the SEZ are tumor precursors or the result of cell migration during cancer evolution (19).

The technique of fluorescence-guided surgical resection was used to isolate matched tumor bulk and SEZ samples from 14 GB patients in order to study the levels of gene expression and copy number variation between the tumor bulk and the SEZ harboring the TICs (10, 19). Cells isolated from the SEZ were found to express increased levels of glial fibrillary acidic protein (GFAP) and the angiogenesis marker CD31 when compared to cells from the tumor bulk, which supports the idea that these cells may have greater replicative potential (19). Interestingly in this study, seven of the nine SEZ samples used for gene expression analysis were classified as mesenchymal GBs (the other two were described as classical), with six of the nine SEZ samples being classified into a different group than the corresponding tumor bulk (19). We statistically validated that the SEZ has differentially expressed genes compared to tissue obtained from the tumor bulk (*p* < 0.00001) and may represent a proliferative center as a result of tumor diversification (19).

Tumor-initiating cells isolated from the SEZ may contribute to therapy resistance, as cells in the SEZ and the tumor bulk continued to proliferate at supramaximal doses of temozolomide while cells were cultured in media that preserved the initial disease genotype (19).

## Neural Networks and Their Use in GB Analysis

**Figure 2:**
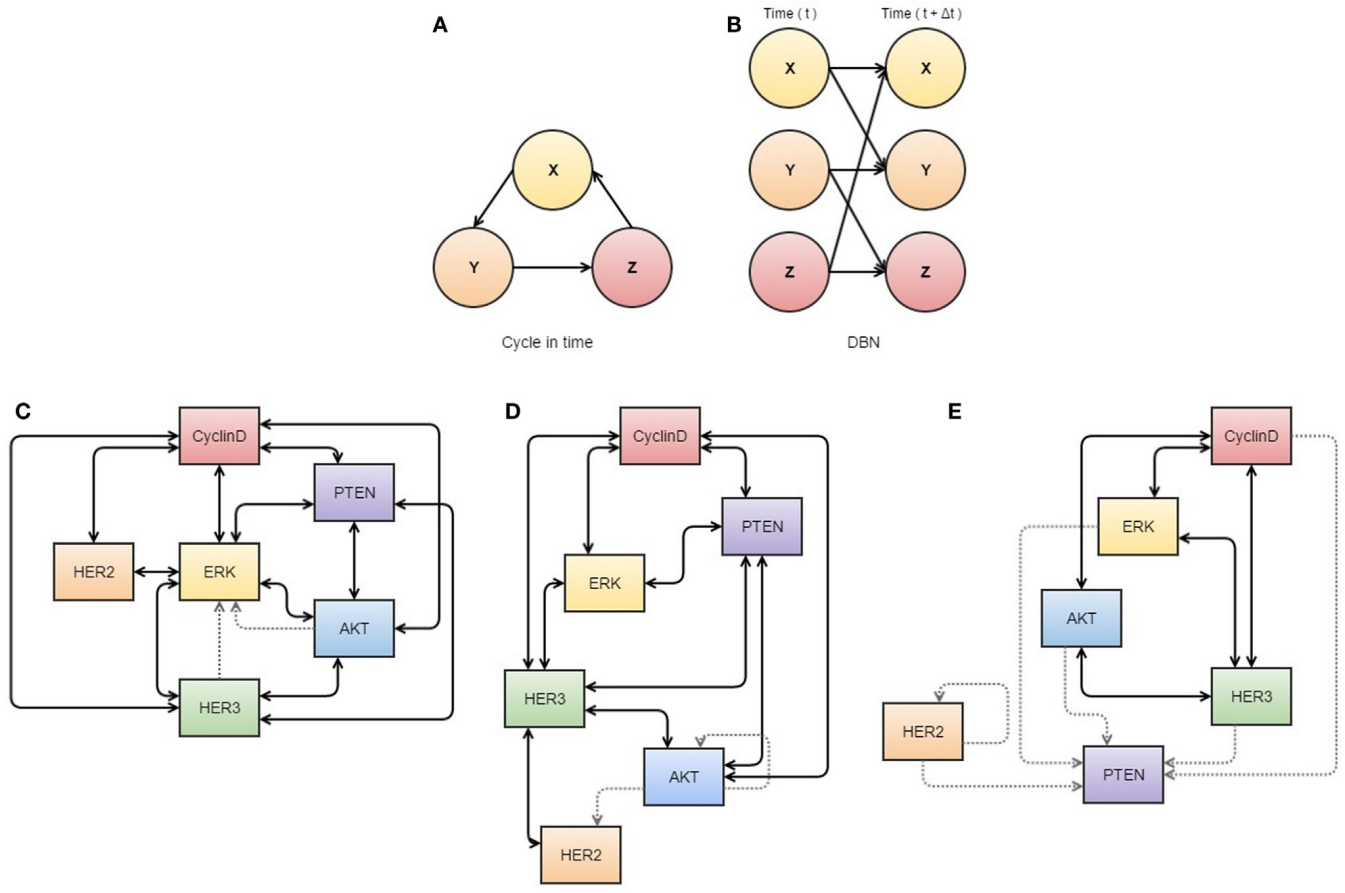
**Neural networks used in cancer systems biology**. **(A,B)** illustrate the use of dynamic Bayesian networks. **(A)** describes how events interact in a cyclic causal manner, as displayed in a dynamic Bayesian network **(B)** that represents all variables at two time points, allowing inference of causal relationships. **(C–E)** are visualizations of S-systems’ analyses. The data represents breast cancer cells treated with heregulin concurrently with pertuzumab (a HER2 inhibitor). The thickness of the lines indicates the strength of the interaction. **(C)** represents the first three time points at which data was obtained, **(D)** the next three overlapping time points, and **(E)** a further three overlapping time points. These diagrams may assist analysis of interactions in the network, as they appear to show dissociation of HER2 in response to treatment with its inhibitor. All components of this figure, including Bayesian networks and S-systems’ analysis, are adapted from the review by Faratian et al. (31).

While no single algorithm can be used to explain the complexity of tumor evolution over time, several theoretical methodologies now exist which may be able to predict tumor response to therapy (31) (see Figure 2).

### Neural Networks and Bayesian Networks

Artificial neural networks are analytical models commonly used to solve classification problems, consisting of nodes and edges that are informal analogs of neurons and synapses in the brain (32–35). External, or visible, nodes correspond to meaningful physical quantities and are divided into input and output nodes (36). Between these two sets of visible nodes lies a large network of hidden nodes, which are not attributed physical significance but act only as a structure for relating the inputs and outputs (36).

The flexibility of this structure is simultaneously a neural networks greatest strength and weakness. With enough data and computing power, neural networks have recently been shown to exhibit extraordinary predictive power and to learn from data without being informed of, or constrained by, causal structures supplied by experts in the system being analyzed (37). Conversely, this flexibility also provides great potential to over-fit to noise or otherwise irrelevant features in the data presented to it (37). Furthermore, without a notional causal model structure with which to compare our system knowledge, or another large dataset to validate against, a fitted neural network may be very difficult to apply sanity-checks to.

So while neural networks certainly constitute an exciting field of quantitative inference, it is also one we should be cautious about. When data are plentiful and the costs associated with incorrect predictions are relatively low, then a neural network may be of great utility – potentially finding model structures modelers had not even imagined or could write down. When data are not plentiful, however, and we require our model to comply with the knowledge of domain experts so that we can more confidently defend its predictions, a Bayesian network model is arguably the more appropriate tool (38). Like a neural network, these models consist of nodes and edges but tend to be much smaller and more rigidly specified (see Figure 2) (36, 38). Here, internal nodes are present only if they, and their connections to other nodes, can be reconciled with mechanisms in the system under consideration (38). Accordingly, the literature on Bayesian networks places significant emphasis on rigorous understanding of a network’s structural properties both in terms of computation and epistemological significance.

A glimpse of the power of cutting-edge neural networks is available in the recent *Nature* review by LeCun et al. (37), while an interesting discussion on their place in statistical learning can be found in the considerably older, but still relevant, Cheng and Titterington (39) and in the responses to that paper. An exploration of the mathematical richness of even quite simple Bayesian networks can be found in Koski and Noble (38), while an advanced introduction to neural networks and related models is provided by Mackay (36).

### Biological Application of Mathematical Models

Kinetic modeling using both experimental and mathematical data can now be used to assess tumor biology over time (31). Some neural networks have been in place clinically for several years for the conversion of MRI data into a three-dimensional tumor landscape in order to determine a target area for radiotherapy (40, 41).

The ultimate aim of these neural networks is to provide a methodology that can be used to convert biomarker data (and associated aberrant pathway signaling) into a treatment regime, based on a predicted outcome (31). If this were the case, the true nature of tumor biology may be identified, allowing a reduction in the use of inferred cancer dynamics from biomarker analysis (31). However, the capacity of any such mathematical model means it is unlikely to be able to describe all parts of the network over space and time due to the amount of biological variation present (31). In order to overcome this, different types of model must be used to analyze different aspects of tumor biology.

### Bayesian Networks and S-Systems to Predict Molecular Interactions

Process-driven modeling allows analysis of molecular interactions between some known pathway components to make mechanistic predictions and evaluate possible outcomes from applying specific pathway inhibitors (see Figure 2) (31). These models have been clinically applied with some success in the case of RTK-inhibitor application for patients with HER2 expression (31). Generally HER2 amplification status is of poor predictive value and not a sufficient predictor of response (42, 43), but a process-driven model has been applied to describe the interactions between inhibitor-receptor binding, HER2/HER3 inhibition, and the regulatory role of PTEN, all in the context of MAPK/PI3K pathway (31). This model was used to determine that PTEN has an important role in resistance to RTK-inhibitors depending on the ratio of PTEN to activated PI3K, and that if PTEN was to be accurately assessed in a clinical setting, it may be used to stratify patients for adjuvant therapies including HER2 inhibitors (31, 44).

Other pathways in cancer are still in need of further elucidation, and it is in these cases when biological knowledge is limited that data-driven modeling can be useful for describing some molecular interactions (31). Bayesian networks (see Figure 2A) can be used to differentiate between direct and indirect relationships of a wide variety of data sets (45), although causality cannot be confirmed (46) unless a time variable is available, which is referred to as a dynamic Bayesian network (Figure 2B) (47). Similarly, S-systems can also be used to fit data in a time-dependent manner to construct a network of interactions for given variables with data sets (31). In application, Biochemical Systems Theory means that S-systems can be used to visualize which links are most susceptible to affecting changes within the system as a whole and therefore can be used to determine new drug targets or identify tumor-suppressor nodes (see Figures 2C–E) (31, 48).

### Mathematical Models in Cancer Evolution and the Cancer Stem Cell Model

Mathematical models are becoming increasingly used in the prediction of cancer initiation and progression (49). The cancer stem cell model was initially developed to describe the dynamics, therapeutic response, and progression of myeloid leukemias, such as CML and APL, but the concept has since been expanded to solid tumors (50, 51). Tumors modeled using the cancer stem cell model have been found to more accurately represent the heterogeneity and invasiveness of human cancer when compared to tumors without the cancer stem cell hierarchy (52, 53). Bayesian networks have previously been used to model melanoma oncogenesis but were ultimately deemed expensive and too complex to interpret (54). Branching processes have also been used to demonstrate the efficacy of combinatorial chemotherapy by analyzing the probability of mono- and combination therapy efficacy (55).

### Neural Networks for GB Analysis

At present, there is no artificial neural network implemented for clinical glioma diagnosis, as there is no single commercial gene signature currently available, although several studies have described differences between glioma and normal brain tissue (56, 57). The study by Mekler et al. aimed to demonstrate the ability of artificial neural networks to cluster gene-expression data from GB and normal brain into two subgroups based on their pathology (56). The overall classification error for the training set was 0.96%, with only 1/160 misclassified normal as GB (0.6%) and 1/48 GB misclassified as normal (2.1%) (56). The subsequent validation set yielded 0% classification error with 44/44 cases correctly classified (56).

Genetic-optimized neural networks have recently been used to predict glioma by reducing the background signal in “noisy” MRI scans in order to allow a more accurate description of tumor location (57). These algorithms are used to cluster pixels and create a mean pixel image to increase resolution and may soon be integrated into medical-imaging systems, though at present they remain a study tool (58).

More recently, Scribner et al. used mathematical modeling to make clinical predictions regarding cell migration in patients treated with Bevacizumab, in an attempt to describe how GB can evade antiangiogenic therapies (59). This can be done by tracking healthy, proliferative, invasive, and necrotic cells using various equations to describe cell behavior in conditions of hypoxia, with further six equations used to monitor angiogenic activity (59, 60). The model was shown to accurately replicate the growth pattern seen in patient scans (59).

Discrimination of driver mutations from late mutations can be made easier by the use of neural networks to assess the effect each component has on the selection pressures active within the system, allowing the identification of targets for molecular therapies (2, 31, 48). However, the application of neural networks to cancer biology is limited due to the lack of adequate temporal resolution that can be achieved using human disease as a model (31).

From an evolutionary perspective, it may be perceived that the diversification of the tumor bulk into multiple tumor subpopulations could be responsible for treatment failure by harboring residual disease and may ultimately be the cause of GB recurrence (19). Further insight into the evolutionary dynamics and signaling pathways of GB may lead to the utilization of molecular therapies targeting multiple tumor subpopulations, with the aim of managing brain cancer as if a chronic disease.

## Models for Predicting Tumor Resistance

At present, it is relatively easy to screen tumor biopsies for several genetic mutations, but the incorporation of this information into treatment strategy formation requires highly accurate and reproducible genomic profiling of tissue samples (2). This could also help optimize the design of clinical trials, and targeted therapies could be greatly beneficial in rare tumor types in which randomized large-scale studies can be impractical (2).

### OncoMap

In 2009, MacConaill et al. developed an optimized mutation profiling platform “OncoMap” in order to evaluate ~400 mutations in 33 known oncogenes and tumor suppressors in 903 assorted frozen and FFPE tumor samples, from 12 various tissue sites (61). Creation of this list of mutations involved mass spectrometry for the initial genotyping, followed by automated base-calling and manual validation, thus use of the OncoMap platform to generate mutation data takes around 7–10 days providing all reagents are in place (61). Many of the genes studied using this profiling system had predictive value for targeted molecular therapies, either as a sensitizing mutation (such as EGFR mutations in NSCLC dictating sensitivity to gefitinib) or as resistance-conferring mutations [such as KRAS mutations in lung and colorectal cancers (62) and PTEN loss in GB which can be used to predict erlotinib resistance (63, 64)].

The specificity of the OncoMap platform operated at up to 100% efficiency in fresh frozen samples and up to 99.4% in FFPE tissue (61). The platform found actionable mutations in 335/903 (37%) of samples across all cancer types (61). PDGFRA and PIK3CA mutations were identified in pediatric low-grade gliomas which could be used to predict response to imatinib/nilotinib or PI3K inhibitors to refine disease prognosis (61). Anticipated frequencies of mutations, such as TP53 and PTEN, were observed in both fresh frozen tissues and FFPE samples (61), which highlight the potential for application of such NGS platforms for use in a clinical setting.

### Using Liquid Biopsies to Predict Tumor Resistance

More recently, the development of technology to isolate circulating tumor DNA has allowed analysis of DNA released from cancer cells into the blood plasma (65). This non-invasive form of liquid biopsy was carried out in six patients by the Rosenfeld group to track mutational changes in the evolution of various metastatic cancers over 1–2 years (65). The examination of breast, ovarian, and lung cancers meant that this study could span multiple treatments at several time points, describing a range of mutant alleles which increased in abundance (allele fraction) with the appearance of therapy resistance (65). Activating mutations in PIK3CA and truncations in RB1 and MED1 was identified, as well as a splicing mutation in growth factor GAS6 and a resistance-conferring mutation in EGFR (65).

This study acts as proof-of-principle that next-generation sequencing techniques can be used to analyze circulating tumor DNA as a non-invasive method of monitoring cancer evolution, as the data from this study (combined with other recent publications) show that the copy number variations and somatic mutations identified in this manner are representative of the tumor genome (65–67). However, a more recent study found that <10% of gliomas patients harbor detectable ctDNA, potentially limiting the application of this powerful methodology to GB (68).

Recently, several studies have successfully isolated circulating tumor cells (CTCs) from peripheral blood and CSF for GB and diffuse glioma, which could yield great potential for disease monitoring to guide treatment (69–76). Sullivan et al. used RNA-*in situ* hybridization to identify a subset of highly migratory tumor cells in GB with a mesenchymal phenotype (69). This led to the conclusion that there may be a subset of mesenchymal cells present in disseminated GB that have the ability to invade the vascular system and proliferate outside the brain as systemic lesions (69). The pilot study by MacArthur et al. employed an assay that can detect the increased telomerase activity in tumor cells using an adenoviral detection system, which is of great benefit as glioma cells oftentimes do not express the molecular markers (such as EpCAM) that are regularly required for CTC assays (71). The MacArthur study identified circulating glioma cells in 8 of 11 (72%) preradiotherapy patients, compared with 1 of 8 (8%) postradiotherapy patients, demonstrating the ability of the liquid biopsy to identify patients at risk of recurrence/with high tumor burdens (71). A larger study by Müller et al. identified CTCs in 29/141 (20.6%) of GB patients by immunostaining for GFAP (70). In this case, the use of a molecular biomarker for CTC isolation was supported by its absence in control participants, and the presence of EGFR amplifications in the tumor cells isolated using GFAP (70). The mobilization of CTCs in the peripheral blood appears to correlate with EGFR amplification that supports the hypothesis that these cells have growth potential (70).

In addition, preliminary studies have identified CTCs in cerebro-spinal fluid using mass spectrometry peptidomics to screen samples for elevated levels of single peptides linked to disease (77). In preliminary data, it appears that the sensitivity of this method allows 4 peptides associated with GB to be identified out of more than 2000 CSF peptides, potentially raising the possibility that lumbar puncture may be able to identify CTCs for disease monitoring (77).

## Conclusion

In conclusion, recent dramatic technological advances in the next-generation sequencing have significantly improved our understanding of intratumour heterogeneity and disease recurrence in GB. Both algorithmic mathematical models, including neural networks and experimental strategies such as multisampling tumor resection, and genetic analysis of circulating cancer cells may in the future help predict the nature of residual disease and resistance to molecular therapies in GB.

However, current challenges lie in the extent of biological variability and the ability of bioinformatics to be able to successfully translate the vast amounts of data generated into a clinically applicable format.

## Conflict of Interest Statement

The authors declare that the research was conducted in the absence of any commercial or financial relationships that could be construed as a potential conflict of interest.
